# Similar Occurrence of Febrile Episodes Reported in Non-Atopic Children at Three to Five Years of Age after Prebiotics Supplemented Infant Formula

**DOI:** 10.1371/journal.pone.0129927

**Published:** 2015-06-15

**Authors:** Margriet van Stuijvenberg, José Stam, Christoph Grüber, Fabio Mosca, Sertac Arslanoglu, Gaetano Chirico, Christian P. Braegger, Josef Riedler, Günther Boehm, Pieter J. J. Sauer, Multicentre Immuno Programming Study (MIPS) Study Group

**Affiliations:** 1 Department of Paediatrics, Beatrix Children’s Hospital, UMC Groningen, Groningen, The Netherlands; 2 Department of Pediatric Pulmonology and Immunology, Charité Universitätsmedizin Berlin, Berlin, Germany; 3 NICU, Fondazione IRCCS Ca’ Granda Ospedale Maggiore Policlinico, Università degli Studi di Milano, Milan, Italy; 4 Centre for Infant Nutrition, Macedonio Melloni Hospital, Milan, Italy; 5 Department of Neonatology, Spedali Civili, Brescia, Italy; 6 Division of Pediatric Gastroenterology and Nutrition, University Children’s Hospital, Zürich, Switzerland; 7 Schwarzach Hospital, Schwarzach, Austria; 8 Danone Nutricia Research, Utrecht, The Netherlands; TNO, NETHERLANDS

## Abstract

This is a follow up study of a multicenter randomised placebo-controlled trial in seven centres in five West European countries. The RCT assessed the effect of infant formula supplemented with a mixture of prebiotics (with neutral short-chain and long-chain oligosaccharides and pectin-derived acidic oligosaccharides) during infancy in term-born children (n=1130). In the follow-up study 672 children (60% of the study population) participated: 232 (56%) from the prebiotics group (PG), 243 (58%) from the control group (CG), and 197 (66%) from the non-randomised breast-fed group (BG). The primary outcome was the occurrence of febrile episodes at three to five years of age prospectively documented by the parents: in the PG 1.17 (interquartile range 0.50-2.08) episodes per year versus 1.20 (0.52-2.57) in the CG; and 1.48 (0.65-2.60) in the BG. This specific prebiotics mixture given during infancy in healthy non-atopic subjects does not decrease febrile episodes and therefore seems not to prevent infection between their third and fifth birthday.

## Introduction

Reduction of infections is one of the major health advantages of breastfeeding compared to formula feeding. [1] Human milk oligosaccharides may contribute to this effect substantially as they have been implicated in the development of the gut flora of breastfed infants. [2–4] Supplementation of infant milk formula with oligosaccharides might represent an option to modulate the intestinal microbiota of formula fed infants as well as acting as immunomodulatory substances and supporting systemic processes contributing to innate defence. *In vitro* studies show an immunomodulatory effect of human milk oligosaccharides as demonstrated by maturation and cytokine production of cord blood T-cells and by influencing various developmental stages in intestinal cells. [5–6] Mouse model studies show an effect on the vaccination response and on the number of lactobacilli in the coecum of oligosaccharide-induced immune modulation, suggesting a role for microbiota. [7] Another study in mice showed a reduction of pulmonary infections in mice fed oligosaccharides. [8] Several clinical studies in human infants have shown a lower incidence of infections when fed with formula to which a combination of probiotics and prebiotics was added. [9–11] The effect of supplementation of prebiotics alone was studied by Arslanoglu et al, who found a lower incidence of respiratory infections and diarrhea in children up to two years of age after receiving prebiotics during the first 6 months of life. [12] In contrast with these findings,our randomised controlled trial comparing the number of fever episodes in healthy term born infants who received either a prebiotic mixture or placebo did not find a decrease in febrile episodes during the first year of life after receiving prebiotics. Instead we found a small decrease in such episodes during the first six months, during which the exposure to prebiotics was highest. [13] Stool consistency was softer in the prebiotics group and closer to that of the breastfeeding group and no difference in adverse events in the two formula groups was found. [14]

Although we found limited results of efficacy in the first year of life, we hypothesized that a long term beneficial effect might exist. An effect after two years was found by Arslanoglu et al. [12] A large population-based infant feeding study showed a reduced number of infections in children at 7 years of age associated with the use of breastfeeding. [1] To date, no study has assessed the effect of supplementation with prebiotics given during the first month of life on infections in children older than two years, i.e. more than a year after the exposure to prebiotics has stopped. We assessed the long term effect of dietary supplementation with prebiotics (6.8 g/L neutral and 1.2 g/L acidic oligosaccharides) to infants in the first year of life on the occurrence of infections in these children at three to five years of age.

## Patients and Methods

We followed healthy full term born infants, who participated in a randomised, double-blind, placebo controlled, multicentre trial (MIPS1), from infancy to five years of age. This study was registered with the German Clinical Trials Register DRKS00000201. Infants randomised to the PG received a standard non-hydrolysed cow’s milk-based formula to which a mixture of neutral short-chain galacto-oligosaccharides and long-chain fructo-oligosaccharides (6.8g/l, ratio 9:1, Immunofortis; Nutricia Cuijk BV, Cuijk, The Netherlands) and pectin-derived acidic oligosaccharides (1.2 g/l) had been added. Details of the RCT (n = 1130) have been published elsewhere. [13] All together 1001 children completed the RCT (MIPS1).

### Procedures in the RCT (MIPS1)

We included healthy full term infants (37 to 42 weeks of gestational age) with a birth weight above the 10^th^ percentile for gestational age according to locally applicable growth charts.

Infants at risk for atopic diseases, i.e. those with a first degree relative with a history of asthma, allergic rhinitis, or atopic dermatitis, were excluded. Infants with neonatal diseases requiring antibiotic treatment, and infants who had previously received formulae containing prebiotics or probiotics were also excluded.

Mothers who could not or did not fully breast-feed their infants were asked to participate in the study and to be randomised to one of the two formula groups before the child reached eight weeks of age. If the parents gave their consent, randomisation occurred the same day. Thus, randomisation and start of the study formula was done at any time before eight weeks after birth.

Parents were invited to participate with their child in the follow-up study if their child had completed the RCT (MIPS1), and if they had not moved to another area. We did not exclude those children who had received treatment with antibiotics, or those who had received supplementation with prebiotics or probiotics. The follow up study took place from November 2008 to February 2011. The study was approved by all local ethical review boards of the participating hospitals: Ethikkommission, Ethikausschuß 2 am Campus Virchow Klinikum, Berlin, Germany; Medisch Ethische Toetsingcommissie, Universitair Medisch Centrum Groningen, The Netherlands; Comitato Etico, Ospedale Maggiore Policlinico,Mangiagalli E Regina Elena; Milano, Italy; Ethikkommission für das Bundesland Salzburg, Austria; Ethikkommission am Kinderspital/Kantonale Ethikkommission Zürich, Switzerland; Comitato Etico Indipendente Ospedale Fatebenefratelli e Oftalmico, Azienda Ospedaliera di rilievo nazionale, Milano, Italy; and Comitato Etico, Spedali Civili, Brescia, Italy. Written informed consent was obtained from each parent. The parents and children were unaware of the group allocation, as were the study physicians and study nurses. The latter only evaluated the infants and were not involved in the statistical analysis and interpretation of the data. None of the researchers who were previously involved in the statistical analysis and interpretation of the data for the MIPS1 study report were involved in the evaluation of any follow-up study participant. An episode of fever was the primary outcome. Follow-up was from the children’s third to fifth birthday. The parents were contacted every three months. At the time of inclusion they were given a diary and were asked to indicate on a form in the diary every day whether their child had fever, and to also report coughing, wheezing, runny or blocked nose, vomiting, diarrhoea, and other symptoms. Fever was defined as a peak rectal temperature of ≥ 38.5°C. We also asked the parents to indicate whether the child had received vaccinations, and whether they had been given antibiotic and antipyretic treatment.

A sample size was not calculated; we included as many children as possible from amongst those who completed the MIPS1 study.

The statistical analyses were performed with SAS, Version 9 (SAS Institute, Cary, NC). All analyses were performed using two-sided testing. Significance level for all testing was 0.05. Per centre the analyses were performed with stratification. The primary analyses were performed using the full analysis set consisting of all children who had been randomised in the PG or the CG, or who had been included in the BG (non-randomised) in MIPS1 according to the intention to treat principle. The PG group and the CG group consisted of children who had received the randomised study formula exclusively up to the age of 20 weeks as well as those who had received the study formula plus breastfeeding. The BG group consisted of children who had received breastfeeding exclusively up to the age of 20 weeks as well as children who had received breastfeeding plus supplemental formula feeding according to the local standards, according to the parents’ preference. Table 1 shows the number of patients included in the MIPS follow up study and attrition during the study period (the first 16 weeks of life) of the groups receiving exclusive study formula and exclusive breastfeeding. At eight weeks of age, 70.7% of all randomised infants in PG and 67.1% in CG received exclusive study formula. These percentages increased up to 84.9% and 84.8%, respectively. At eight weeks 92.9% of all children in BG received exclusive breastfeeding, decreasing to 83.8% at 16 weeks.

**Table 1:** Exclusive study formula and exclusive breastfeeding per 2 week time point in the first 16 weeks of life.

Week	PG (n = 232)	CG (n = 243)	BG (n = 197)
8	164 (70.7%)	163 (67.1)	183 (92.9)
10	187 (80.6%)	198 (81.5)	171 (86.8)
12	189 (81.5%)	206 (84.8)	178 (90.4)
14	196 (84.5%)	201 (82.7)	174 (88.3)
16	197 (84.9%)	206 (84.8)	165 (83.8)

PG—Prebiotics Group; CG—Control Group; BG—Breast-fed Group

The primary outcome variable was defined as: (frequency of fever episodes + 0.05) divided by (duration of observational period in days + 0.1). This ‘adjustment’ was done in order to avoid ‘zero’ for those cases with no episode of fever. Furthermore, by this ‘adjustment’ the duration of the observational period, including the observational period of those cases with zero fever episodes could be taken into account. [15] The observational period (which is the time at risk in which the number of fever episodes was counted) started at inclusion in the MIPS follow up study which was the third birthday. To present the frequency of fever episodes per year, frequency was multiplied by 365.1 days. By using this technique the frequency of fever episodes per child could be calculated. Similar calculations were done for the frequency of episodes with coughing, wheezing, runny or blocked nose, vomiting, diarrhoea, and other symptoms. We also calculated the duration of these symptoms. Differences in the frequency of symptoms per year and in the duration of symptoms in the PG versus the CG group; and the CG versus the BG group, were analysed for all centres using the non-parametric Van Elteren test. This is a stratified Wilcoxon rank sum test. Results are presented in median values with their 25 and 75 percentiles. Differences between groups with respect to baseline characteristics, vaccinations, and the use of antibiotics and antipyretics were analysed using a non-parametric test (two-sided, Mann Whitney).

## Results

**Fig 1:**
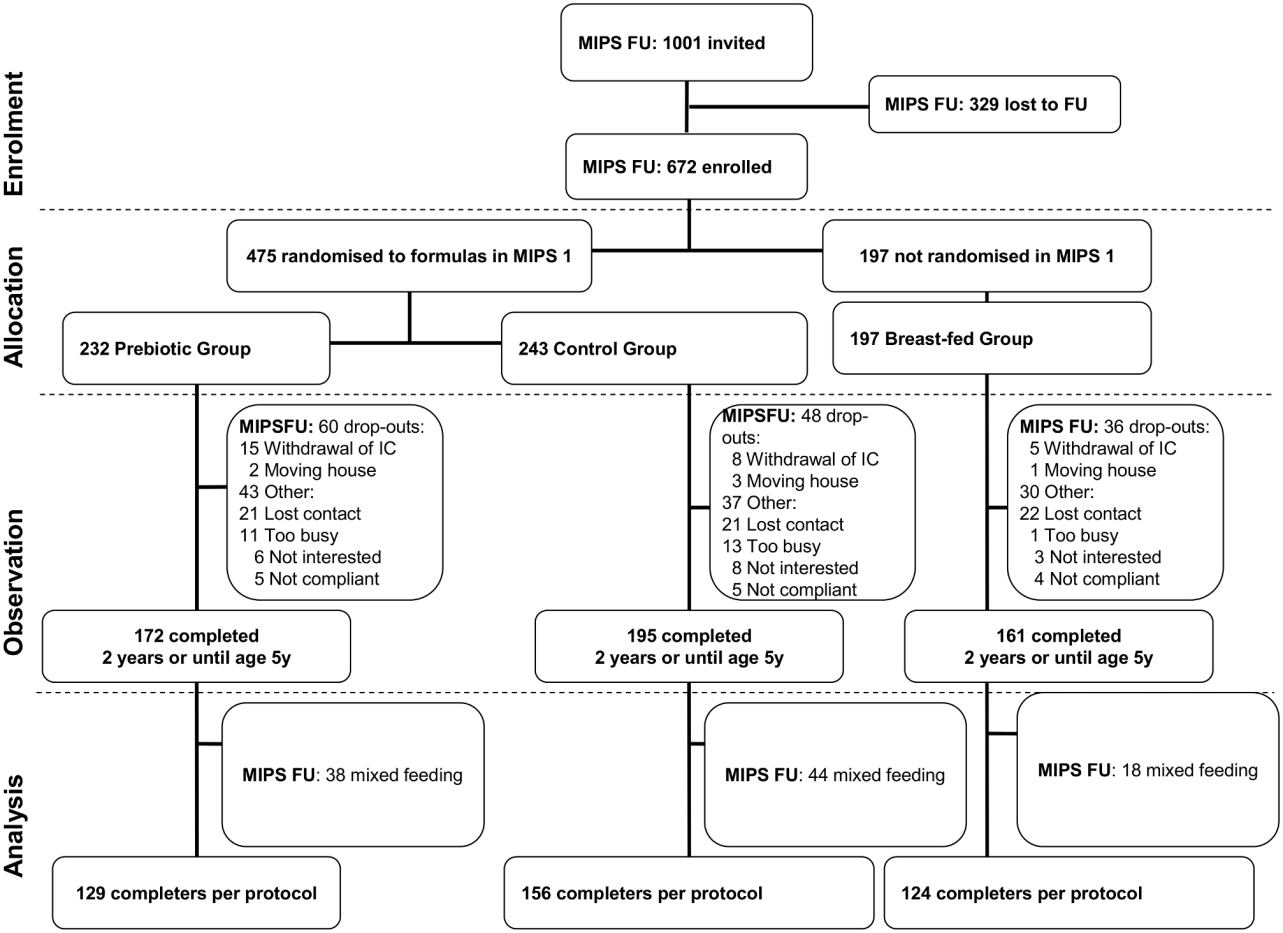
Flow diagram.

We included 672 (60% of the 1130 participants in MIPS1) in the MIPS follow up study. Of the original study population (n = 1130), 458 children were not included in the follow-up study: 129 children did not complete the RCT (MIPS1), and another 329 children could no longer be contacted because they had moved to another area or had changed their contact information. We included 232 (56% of the 414 participants in this group in MIPS1) from the PG, 243 (58%) from the CG, and 197 (66%) from the BG (Fig 1).

Differences in baseline characteristics, including those which may be associated with the outcome, were compared within the treatment groups (PG and CG) between the original MIPS1 study population versus the MIPS follow up study population. The only statistically significant difference we found was in the number of rooms in the house in the PG (MIPS1 (n = 414) 3 (3–4) rooms versus in MIPS follow-up (n = 232) 4(3–5) rooms), and in the CG (MIPS1 (n = 416) 3 (3–4) rooms versus in MIPS follow-up (n = 243) 4 (3–4) rooms). This was similar in the Prebiotics Group and the Control Group.

The baseline characteristics at the beginning of the MIPS follow-up study are presented in Table 2. We found no statistically significant differences between the PG and the CG. Median age of inclusion in the RCT was 30 days of life for the prebiotics group (PG), 32 days for the control group (CG), and 50 days for the breast fed group (BG). Table 3 shows the data about the vaccination pattern in the PG, CG, and BG. There were no statistical differences between the PG and CG.

**Table 2:** Baseline characteristics.

	Prebiotics Group (PG) (n = 232)^2^	Control Group (CG) (n = 243)^2^	Breast-fed Group (BG) (n = 197)^2^	PG vs CG^1^ *P* value
Children				
Age at inclusion (days)	30 (19–43)	32 (21–46)	50 (38–54)	0.25
Age at inclusion in MIPS FU (months)	36 (35–36)	36 (35–36)	36 (35–36)	0.42
Birth weight (grams)	3263 (2960–3600)	3290 (2910–3630)	3480 (3160–3700)	0.96
Weight at inclusion (kilograms)	15.0 (13.9–16.1)	14.8 (13.8–16.0)	14.7 (13.8–15.8)	0.35
Boys	113 (48.7)	114 (46.9)	91 (46.2)	0.70
Vaginal delivery	147 (63.3)	154 (63.4)	167 (84.8)	0.99
Number of children in household	2 (1–2)	2 (1–2)	2 (2–2)	0.72
Parents				
Smoking mother				
-Before pregnancy	80 (34.6)	80 (33.1)	57 (28.9)	0.72
-During pregnancy	32 (13.9)	26 (10.7)	14 (7.1)	0.30
-Since child’s 1st birthday	58 (25.1)	56 (23.1)	31 (15.7)	0.62
Smoking father				
-Since child’s 1st birthday	72 (31.4)	84 (35.4)	48 (24.5)	0.36
Preschool admission				
-At 3 years of age	121 (52.2)	127 (52.3)	101 (51.3)	0.98
-At 5 years of age	126 (54.3)	131 (53.9)	119 (60.4)	0.24
Education level of the mother				0.47
-Primary and secondary school	74 (31.9)	82 (33.8)	56 (28.4)	
-Some university education/post-secondary education/technical or trade qualification	114 (49.1)	106 (43.8)	92 (46.7)	
-Completed university degree	44 (19.0)	54 (22.3)	49 (24.9)	
Mother occupational status				0.07
-Employed	157 (67.7)	166 (68.9)	134 (68.7)	
-Self-employed	16 (6.9)	26 (10.8)	13 (6.7)	
-Seeking work/Student/Unemployed	11 (4.7)	3 (1.2)	5 (2.6)	
-Not working	48 (20.7)	46 (19.1)	43 (22.0)	
Education level of the father				0.21
-Primary and secondary school	78 (34.4)	71 (29.7)	41 (21.0)	
-Some university education/post-secondary education/technical or trade qualification	106 (46.7)	107 (44.8)	100 (51.0)	
-Completed university degree	43 (18.9)	61 (25.5)	55 (28.0)	
Father occupational status				>0.99
-Employed	176 (77.5)	184 (77.3)	138 (70.8)	
-Self-employed	43 (19.0)	45 (18.9)	47 (24.1)	
-Seeking work/Student/Unemployed	7 (3.1)	8 (3.4)	8 (4.1)	
-Not working/Disabled	1 (0.4)	1 (0.4)	2 (1.0)	

^1^ Non-parametric test (PG versus CG)

^2^ Median (interquartile range) or n (%)

**Table 3:** Vaccination data: number and percentage of children who received vaccinations before inclusion of MIPS Follow Up and during MIPS Follow Up,

	Prebiotics Group (PG) (n = 232)	Control Group (CG) (n = 243)	PG vs CG (*P* value)^1^	Breast-fed Group (BG) (n = 197)	CG vs BG (*P* value)^1^
Before inclusion in MIPS FU (at baseline)					
-BMR	207 (89.2%)	216 (88.9%)	0.91	174 (88.3%)	0.85
-D(K)TP	10 (4.3%)	13 (5.3%)	0.60	12 (6.1%)	0.74
-D(K)TP plus HIB	101 (43.5%)	90 (37.0%)	0.15	110 (55.8%)	<0.01
-HIB	11 (4.7%)	16 (6.6%)	0.39	3 (1.5%)	<0.01
-Meningococ C	118 (50.9%)	121 (49.8%)	0.82	89 (45.2%)	0.34
-Pneumococ	70 (30.2%)	77 (31.7%)	0.72	74 (37.6%)	0.20
-Miscellaneous	27 (11.6%)	34 (14.0%)	0.44	42 (21.3%)	0.04
From inclusion to 4th birthday					
-BMR	3 (1.3%)	1 (0.4%)	0.29	4 (2.0%)	0.11
-D(K)TP	45 (19.4%)	50 (20.6%)	0.75	30 (15.2%)	0.15
-D(K)TP plus HIB	1 (0.4%)	0 (0%)	0.31	0 (0%)	1.00
-HIB	49 (21.1%)	57 (23.5%)	0.54	38 (19.3%)	0.29
-Meningococ C	9 (3.9%)	3 (1.2%)	0.07	8 (4.1%)	0.06
-Pneumococ	1 (0.4%)	3 (1.2%)	0.34	0 (0%)	0.12
-Miscellaneous	13 (5.6%)	14 (5.8%)	0.94	18 (9.1%)	0.18
From 4rth birthday to 5th birthday					
-BMR	1 (0.4%)	0 (0%)	0.31	4 (2.0%)	0.03
-D(K)TP	11 (4.7%)	12 (4.9%)	0.92	4 (2.0%)	0.11
-D(K)TP plus HIB	0 (0%)	1 (0.4%)	0.33	1 (0.5%)	0.88
-HIB	2 (0.9%)	4 (1.6%)	0.45	2 (1.0%)	0.57
-Meningococ C	2 (0.9%)	4 (1.6%)	0.45	7 (3.6%)	0.20
-Pneumococ	0 (0%)	1 (0.4%)	0.33	3 (1.5%)	0.22
-Miscellaneous	6 (2.6%)	6 (2.5%)	0.94	13 (6.6%)	0.03

^1^ Non-parametric test (PG versus CG; CG versus BG; PG not tested versus BG)

Table 4 shows the adjusted frequency of fever episodes and other illness symptoms during follow-up. We found no statistically significant differences between the PG and the CG in the occurrence of fever and other illnesses: in the PG 1.17 (median value, interquartile range 0.50–2.08) febrile episodes per year were reported versus 1.20 (0.52–2.57) in the CG (p = 0.22); and 1.48 (0.65–2.60) in the BG. We found a higher frequency of ‘other symptoms’ runny or blocked nose in the BG versus the CG.

**Table 4:** Adjusted frequency of febrile episodes per year from third to fifth birthday, median and 25–75 percentiles.

	Prebiotics Group (PG) (n = 232)	Control Group (CG) (n = 243)	PG vs CG (*P* value)^1^	Breast-fed Group (BG) n = 197	CG vs BG (*P* value)^1^
Fever	1.17 (0.50–2.08)	1.20 (0.52–2.57)	0.22	1.48 (0.65–2.60)	0.42
Coughing	2.55 (1.09–4.07)	2.63 (1.09–4.56)	0.38	3.02 (1.52–4.80)	0.24
Wheezing	0.03 (0.02–0.54)	0.03 (0.02–0.52)	0.18	0.04 (0.02–0.52)	0.09
Runny or blocked nose	2.59 (1.02–5.26)	2.91 (0.99–4.95)	0.93	3.62 (1.97–5.76)	0.05
Vomiting	0.87 (0.04–1.56)	0.91 (0.09–1.55)	0.46	1.00 (0.47–1.93)	0.70
Diarrhoea	0.51 (0.03–1.07)	0.53 (0.03–1.51)	0.22	0.57 (0.04–1.55)	0.53

^1^ Van Elteren test (PG versus CG; CG versus BG; PG not tested versus BG)

The term ‘adjusted’ reflects to the statistical ‘correction’ for zero values for those cases with zero illness episodes.

In Table 5 we present the duration of illness episodes in days. There was a shorter duration of ‘diarrhoea’ in the PG versus the CG (*P* = .01).

**Table 5:** Duration of illness episodes in days from third to fifth birthday, median and 25–75 percentiles

	PG (n = 232)	CG (n = 243)	PG vs CG (*P* value)^1^	BG (n = 197)	CG vs BG (*P* value)^1^
Fever	4.0 (0.0–9.5)	6.0 (1.0–13.0)	0.08	5.0 (2.0–11.0)	0.60
Coughing	22.5 (8.0–42.5)	24.0 (9.0–50.0)	0.27	32.0 (10.0–55.0)	0.70
Wheezing	0.0 (0.0–0.2)	0.0 (0.0–0.2)	0.74	0.0 (0.0–0.3)	0.89
Runny or blocked nose	21.0 (5.0–61.0)	24.0 (6.0–60.0)	0.66	34.0 (10.0–79.0)	0.36
Vomiting	2.0 (0.0–4.0)	2.0 (0.0–5.0)	0.13	2.0 (0.0–5.0)	0.33
Diarrhoea	1.0 (0.0–4.0)	2.0 (0.0–7.0)	0.01	2.0 (0.0–6.0)	0.67

^1^ Van Elteren test (PG versus CG; CG versus BG; PG not tested versus BG)

PG—Prebiotics Group; CG—Control Group; BG—Breast-fed Group

In Table 6 the use of antibiotics is shown, with no statistical significant differences between the two study formula groups. Table 7 shows the use of antipyretics. We found no statistical significant differences between the two study formula groups.

**Table 6:** Use of antibiotics from third to fifth birthday.

Antibiotic type	PG (n = 232)	CG (n = 243)	PG vs CG (*P* value)^1^	BG (n = 197)	CG vs BG (*P* value)^1^
Pheneticilline	3 (1.3%)	3 (1.2%)	0.95	-	0.12
Phenoxymethylpenicilline	5 (2.2%)	11 (4.6%)	0.15	11 (5.6%)	0.61
Amoxicillin plus clavulanic acid	42 (18.1)	61 (25.1%)	0.06	43 (21.8)	0.42
Amoxicillin	47 (20.3%)	47 (19.3%)	0.80	27 (13.7%)	0.12
Sulfonamides plus trimethoprim	2 (0.9)	4 (1.6%)	0.44	-	0.07
Cefalosporines or fluoroquinolones	2 (0.9%)	1 (0.4%)	0.54	1 (0.5%)	0.88
2^nd^ generation cephalosporines	19 (8.2%)	16 (6.6%)	0.50	20 (10.2%)	0.18
3^rd^ generation cephalosporines	21 (9.1%)	23 (9.5%)	0.88	29 (14.7%)	0.09
Macrolides	31 (13.4%)	35 (14.4%)	0.74	19 (9.6%)	0.13
Nitrofurantoine	-	2 (0.8%)	0.17	-	0.20

^1^ Non-parametric test (PG versus CG; CG versus BG; PG not tested versus BG)

PG—Prebiotics Group; CG—Control Group; BG—Breast-fed Group

Number and percentage of children per study group having one or more doses of antibiotics

**Table 7:** Use of antipyretics from third to fifth birthday.

Antipyretic type	PG (n = 232)	CG (n = 243)	PG vs CG (*P* value)^1^	BG (n = 197)	CG vs BG (*P* value)^1^
Paracetamol	157 (67.7%)	165 (67.9%)	0.96	117 (59.4%)	0.06
Ibuprofen or Naproxen	67 (28.9%)	72 (29.6%)	0.86	81 (41.1%)	0.01

^1^ Non-parametric test (PG versus CG; CG versus BG; PG not tested versus BG)

PG—Prebiotics Group; CG—Control Group; BG—Breast-fed Group

Number and percentage of children per study group having received one or more doses of antipyretics

## Discussion

In this study we show that it is unlikely that dietary prebiotic supplementation in the first year of life has an effect on the frequency of febrile episodes in otherwise healthy non-atopic children during their third to their fifth year. We found a reduction of one day of the duration of episodes of diarrhoea (Table 5). The clinical significance of this finding may be limited, although shortening the duration of illness by a day is certainly relevant for parents.

In the MIPS1 study we failed to find an effect of prebiotic supplementation in reducing the frequency of febrile episodes in the first year of life while previously we found a small difference in the frequency of febrile episodes in the first six months in the PG versus the CG (both median values 0.13, P<0.05) in favour of the PG. [13] In an additional post hoc ‘per protocol’ analysis, we used the data of the participants who had either received prebiotic formula or the control formula exclusively for the first four months of life (20 weeks) or who had been breast-fed exclusively for the first four months of life (20 weeks), had completed the MIPS1 until their first birthday, and had completed the follow-up from their third to their fifth birthday only (Fig 1). For this posthoc analysis we assessed the occurrence of febrile episodes between their third to their fifth birthday. No statistically significant difference in the PG versus the CG was found. We may speculate that the effect of prebiotics is found only at the time the prebiotics are given, i.e. the period during which the infants received the prebiotics-supplemented formula as the greater part of their nutritional intake. After this period other products increasingly predominate the nutritional intake of the child and the effect of prebiotics may fade away.

Although it might be speculated that the effect of the study formula was limited because this was often combined with breastfeeding, this was, in fact, not the case: 67–85% of children received exclusive study formula feeding (Table 1). However, the frequency of febrile episodes in this healthy non-atopic study population in developed countries may be too low to demonstrate a statistically significant effect: in the MIPS 1 study overall there were 1.2 fever episodes per year, and in the MIPS follow up study 1.2 to 1.5 fever episodes per year. Aside from the low occurrence of the main study outcome (i.e. the frequency of febrile episodes), another limitation of this study is the substantial number of children which were lost to follow up since the start of the RCT (60% of MIPS1 participants were included). The main reason for this is likely to be the relatively long time interval (two years) between the end of the RCT and the start of the follow up study. Additionally, about a third of those not included in the follow up study were children who did not complete the RCT (n = 129). We did a post hoc sample size calculation with the assumption of two febrile episodes per year (and two years of observation) and a reduction of10% in the intervention group (alpha 0.05 and power 0.80, two-sided) resulting in 1203 children per group. These numbers may suggest that the power of the study is limited. However its use is not primarily interpreting data. An indication of the value of the study results may come from the 95% confidence interval limits for the difference between PG and CG which are -0.74 and 0.40, with a mean difference of -0.17 (Least Square Means, ANCOVA). It is very unlikely that the difference between PG and CG is more than one fever episode per year. [16]

If an effect of prebiotic supplementation exists, this is likely to be small and not clinically relevant. We found only one difference in baseline characteristics in the original MIPS1 study population versus the MIPS follow-up study population, but this difference (number of rooms in the household) was similar in the PG and CG. Thus, although the original MIPS1 study group and the current MIPS follow up group differ as to this one baseline characteristic, this is unlikely to affect the validity of the comparison between the PG and CG.

The incidence of reported febrile episodes being lower that expected may at least partly be explained by the relatively very healthy study population (i.e. without a family history of atopic disease). The incidence of febrile episodes also may have been influenced by the fact that the main outcome (febrile episodes) relied on parental reports. However, this outcome is close to everyday practice in the way that illnesses are recognised in families with young children. By prospective documentation (using a diary, repeated telephone calls and visits) any loss of information was further minimalised. An underestimation of the number of febrile episodes may have been caused by the use of rectal temperature measurements since many parents currently prefer the use of a tympanic thermometer. Based on our three-monthly telephone contact with the parents our impression is that for the purpose of the study parents all were highly compliant with all aspects of the study, including the use of a rectal thermometer. We cannot exclude with certainty that no febrile episodes were missed. Data using more objective outcome measures such as GP or pediatrician visits were not collected. Clear and uniform criteria would probably result in the discounting of at least some illness episodes not fulfilling the criteria. Data on the use of antibiotics or antipyretics do not show clinically relevant differences between the two study formula groups (PG and CG). We conclude that an effect of prebiotic supplementation in the first year of life is unlikely to reduce the number of infections at age three to five years.

Our data suggested a higher frequency of ‘runny or blocked nose’ (Table 3) in the non-randomised BG compared to the CG. We found similar results in MIPS1. This difference may be associated with a different parental attitude towards illness of their children in the non-randomised BG. As a group breast feeding parents have a higher socioeconomic and educational level compared to parents who choose to formula feed their child. [17]Recent studies from developed countries demonstrated a clear association of breastfeeding with fewer infections. [18] Problems with controlling for possible confounders such as hereditary and household factors remain. [17]

In this study data about nutritional habits have not been collected. Recent studies showed influence of dietary micronutrient intake on the occurrence of infections. Most of these studies have been done in developing countries [19] in specific patient groups [20], or do not present data about infections [21]. It is difficult to extrapolate these study results to other populations.

In this study we supplemented the infant formula with three oligosaccharides: neutral short- chain galacto-oligosaccharides, neutral long-chain fructo-oligosaccharides, and pectin-derived acidic oligosaccharides. As yet, there is no clarity as to the optimal composition and amount of prebiotics. Thus far, most studies used a combination of short-chain galacto-oligosaccharides and long-chain fructo-oligosaccharides. A study in mice showed a stimulatory effect on the Th1 type immune response when pectin-derived acidic oligosaccharides were added to the mixture. (7) A recent RCT demonstrated that a mixture containing neutral and acidic oligosaccharides added to the milk of breast milk-fed and to the formula of formula-fed preterm babies did not reduce the occurrence of infectious diseases in these infants. [22,23] Differences in the composition of the prebiotic mixtures are small, but it is unknown if these may lead to different results. The results of our study cannot be extrapolated to other prebiotics.

The results obtained in our previous studies and in the present study, do not support the use of this mixture to reduce the frequency of infections in children up to five years of age. This conclusion should be taken with caution because the power of this present study is limited by the sample size and the low frequency of the main outcome parameter.

## Conclusion

Although our study has limited power, it does not support the notion that this specific prebiotics mixture neutral short chain galacto-oligosaccharides and long chain fructo-oligosaccharides 6.8 g/L, ratio 9:1 and pectin derived acidic oligosaccharides 1.2 g/L used in standard formula during infancy prevents infection as it has not decreased febrile episodes in non-atopic children between their third and fifth birthdays.
